# Genetic Evidence Strongly Support an Essential Role for PfPV1 in Intra-Erythrocytic Growth of *P. falciparum*

**DOI:** 10.1371/journal.pone.0018396

**Published:** 2011-03-31

**Authors:** Trang Chu, Klaus Lingelbach, Jude M. Przyborski

**Affiliations:** Department of Parasitology, Faculty of Biology, Philipps University Marburg, Marburg, Germany; University of Bern, Switzerland

## Abstract

Upon invading the host erythrocyte, the human malaria parasite *P. falciparum* lives and replicates within a membrane bound compartment referred to as the parasitophorous vacuole. Recently, interest in this compartment and its protein content has grown, due to the important roles these play in parasite egress and protein traffic to the host cell. Surprisingly, the function of many proteins within this compartment has not been experimentally addressed. Here, we study the importance of one of these proteins, termed PfPV1, for intra-erythrocytic parasite survival. Despite numerous attempts to inactivate the gene encoding PfPV1, we were unable to recover deletion mutants. Control experiments verified that the *pv1* gene locus was *per se* open for gene targeting experiments, allowing us to exclude technical limitations in our experimental strategy. Our data provide strong genetic evidence that PfPV1 is essential for survival of blood stage *P. falciparum*, and further highlight the importance of parasitophorous vacuole proteins in this part of the parasite's life cycle.

## Introduction

Having invaded the host erythrocyte, *Plasmodium falciparum* generates and maintains a parasitophorous vacuole (PV), within which the parasite subsequently replicates [1]. This vacuole represents a unique compartment, shielding the parasite from the host cell cytosol, but nevertheless allowing passage of nutrients, waste products and even large polypeptides both to, and from the host cell and surrounding environment [1]. In recent years interest in the PV and it contents has grown, due to the roles its proteins play in parasite egress and protein traffic to the host cell [2]–[4]. In a recent study, we identified 27 putative PV proteins, including several proteins with no recognisable homology outside of Plasmodium spp [5]. One of the proteins identified in this analysis was termed PfPV1. As no reliable *in silico* data were available to predict the function of this protein, we decided to follow a reverse genetic strategy to investigate the importance of the PfPV1 protein for parasite proliferation and survival. Despite numerous attempts to disrupt the gene encoding PfPV1, we were not able to isolate deletion mutant cell lines. Control experiments targeting the gene locus verified that the *pv1* gene is accessible for genetic manipulation. Our data provide strong genetic evidence for an essential role of PfPV1 in intracellular survival of *P. falciparum*.

## Results

### PfPV1

PfPV1 is encoded by PF11_0302, which is located within a region on chromosome 11 which shares synteny in *P. vivax*, *P. knowlesi* and *P. chaubaudi* [6]. Data mining identify homologues in all Plasmodium species for which high quality sequencing data is available (Table 1, accession numbers refer to PlasmoDB [6]). The ortholog group represented by PfPV1 (OG4_48336) is restricted to Plasmodium spp. PV1 orthologs cannot be detected by manual reciprocal homology based searches even in other apicomplexan parasites such as *T. gondii* or Babesia spp [7]. PfPV1 is slightly larger than homologous PV1 proteins, most of this difference can be attributed to a 42 amino acid QE acid rich insertion towards the C-terminal end of the protein (Fig. S1). Although the function of such regions remains unclear, such QE rich regions are common in *P. falciparum* proteins [8], [9]. Transcriptome and proteomics data suggest that PfPV1 is highly expressed in all stages of both the asexual, and sexual blood stages, and PfPV1 has also been detected in the proteome of parasites isolated from patients [6]. PfPV1 is predicted to contain an N-terminal secretory signal sequence [10], but lacks further hydrophobic regions or recognisable protein sorting motifs. Although various bioinformatic tools predict several structural motifs within the sequence of PfPV1, these algorithms do not detect the same domains when applied to homologous sequences from other Plasmodium species, and are thus likely to represent *in silico* artefacts.

**Table 1:** PV1 homologues.

	*P. falciparum*	*P. knowlesi*	*P. vivax*	*P. chaubaudi*	*P. yoelii*	*P. berghei*
**Acc. No.**	PF11_0302	PKH_092690	PVX_092070	PCAS_092700	PY06925*	PB000970.00.0*
**Length (aa)**	452	428	429	390	283	130
**SS**	Y	Y	Y	Y	N	N
**Syntenic**	-	Y	Y	Y	N	N
**% Identity** #	-	26.2	26.0	22.9	-	-

*Possibly incomplete gene model.

# Compared to *P. falciparum*.

Acc. No., PlasmoDB accession number; SS, signal sequence. Synteny based on *P. falciparum*. % identity calculated against *P. falciparum*. Identity was not calculated for *P. yoelii* or *P. berghei* as it is likely that these are incomplete sequences.

### PfPV1 localises solely to the PV

**Figure 1:**
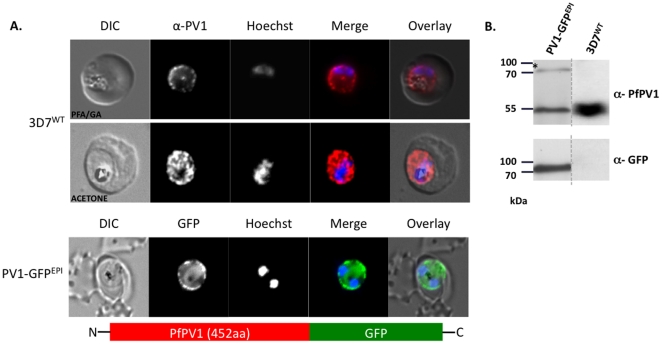
Localisation of PfPV1. (A, upper and middle panel) Immunofluorescence localisation using specific anti-PfPV1 antibodies. (A, lower panel) Episomal expression of a PfPV1-GFP chimera. (B) Western blot analysis on protein extracts derived from PV1-GFP^EPI^ infected erythrocytes. DIC, differential interference contrast; GFP, green fluorescent protein; Hoechst, nuclear staining. GFP, green; specific antibody, red; Hoechst, blue.

Using antibodies specific for the PfPV1 protein, we have previously localised this protein to the parasitophorous vacuole, and also to the Maurer's clefts [5]. These data were slightly confusing, considering that PfPV1 does not contain a PEXEL/HT motif required for trafficking to the host cell [2]. To address this, we carried out further analyses to define the sub-cellular localisation of this protein. Firstly, we carried out immuno-fluorescence using anti-PfPV1 antibodies. We utilised two different fixation protocols. Initially, we applied a modified form of the fixation protocol developed by Tonkin *et al*. [11]. The advantages of this technique over the traditional methanol or methanol/acetone fixation are lower auto-fluorescence, and a much better preserved cell morphology [11]. Under these conditions, specific labelling of PfPV1 can be seen only in a “ring of beads” structure surrounding the body of the parasite, indicative of a PV localisation (Fig. 1a, upper panel). No fluorescent signal could be detected within the erythrocyte. To verify this using an independent fixation protocol, we also carried out immuno-fluorescence using anti-PfPV1 antibodies on acetone-fixed blood smears. Although the “ring of beads” pattern was less well defined than under the initial fixation protocol, importantly we could detect fluorescence only associated with the parasite, with no signal detected in the erythrocyte cytosol (Fig. 1a, middle panel). To substantiate these observations by a further method, we generated transgenic parasites episomally expressing PfPV1 fused to a GFP reporter, under control of the PfCRT promoter. Following transfection into blood stage 3D7 parasites and selection with WR99210, a drug resistant parasite population could be established after 21 days, referred to as PV1-GFP^EPI^. Live cell imaging of erythrocytes infected with PV1-GFP^EPI^ reveals GFP fluorescence in a “ring of beads” around the parasite (Fig. 1a, lower panel). Western blot analysis verifies that the parasites synthesise, in addition to the endogenous PfPV1, a PfPV1-GFP chimera of approximately the expected molecular mass (77 kDa, Fig. 1b, left). As previously demonstrated, when probing proteins extract derived from wild type 3D7 parasites with anti-PV1 antibodies, only one signal could be detected, correlating with the expected size of the endogenous PV1 protein (55 kDa, Fig. 1b, right [5]). As expected, no signal was detected when probing 3D7 protein extracts with anti-GFP antibodies (Fig. 1b). Taken together, these data strongly suggest that PfPV1 is a *bona fide* PV protein.

### The *pv1* locus cannot be targeted by a simple double-crossover knockout strategy

**Figure 2:**
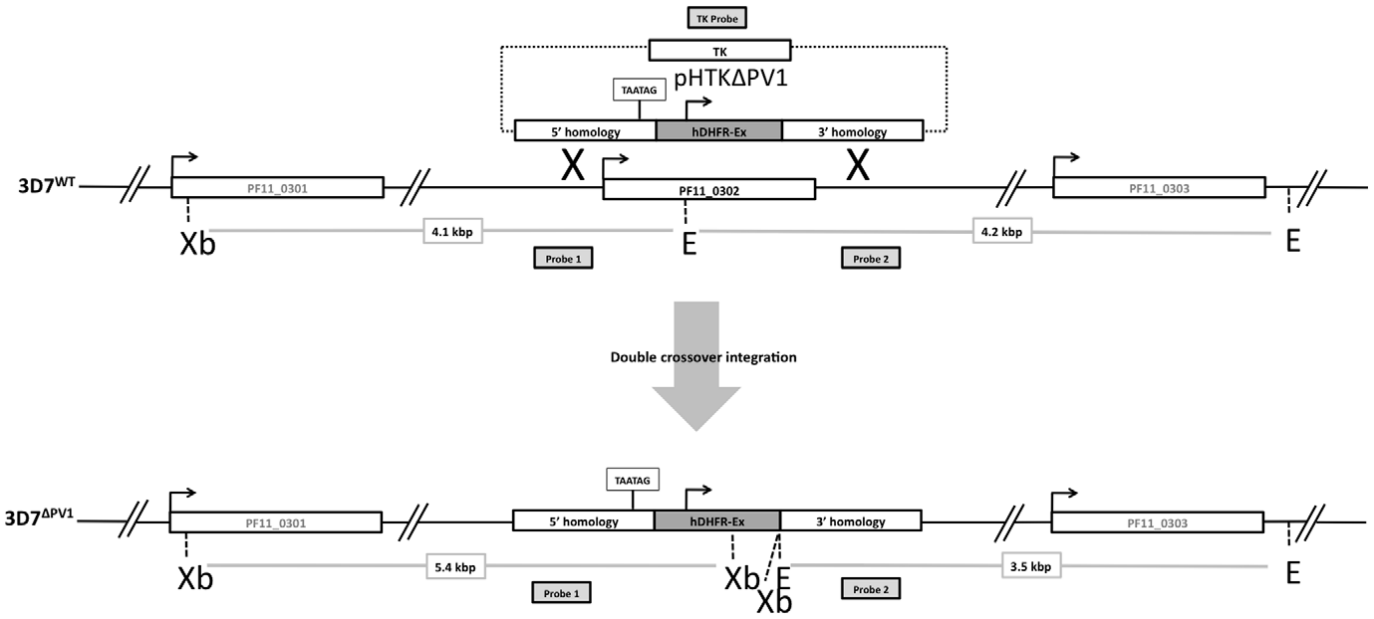
Schematic representation of double crossover integration strategy. Following a successful recombination event, the *pv1* gene would be replaced with the hDHFR selection cassette. Xb, XbaI; E, EcoRI; TK, thymidine kinase. Not to scale. Probes used in subsequent Southern analyses are shown.

To investigate the importance of PfPV1 for blood stage parasites, we generated a knockout plasmid, based on the double-crossover vector pHTK [12]. In this vector (pHTKΔPV1), the selectable marker expression cassette (hDHFR-Ex) is flanked by two regions derived from homologous genomic sequences surrounding the *pv1* locus. Furthermore, the backbone of this vector contains a thymidine kinase (TK) expression cassette, to allow negative selection against unwanted integration events. A schematic of the planned integration events is presented in Fig. 2. Following transfection into blood stage 3D7 parasites and selection with WR99210, a drug resistant parasite population could be established after 21 days, referred to as 3D7^TKEPI^.

**Figure 3:**
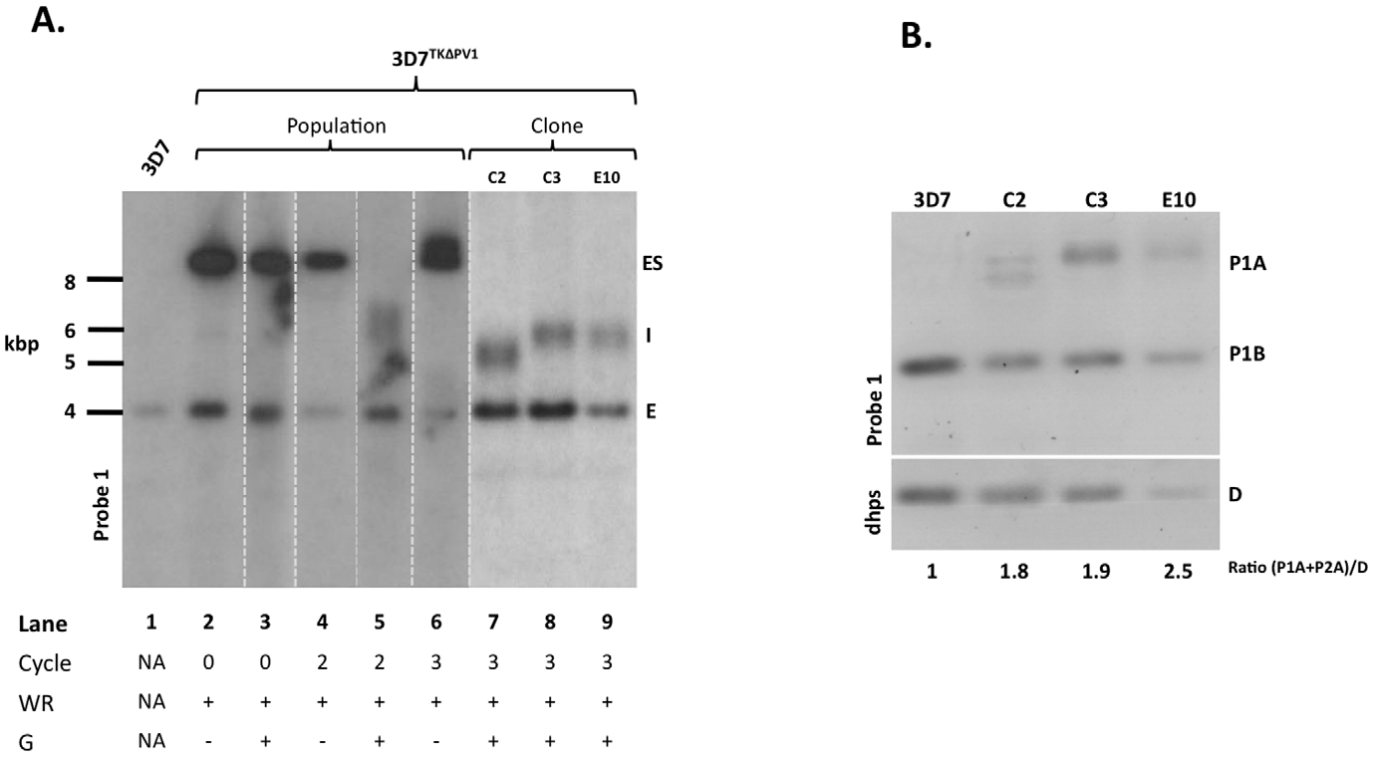
Southern blot analysis I. (A) Southern analysis on XbaI/EcoRI digested gDNA derived from the parasite lines indicated. WR, WR99210; G, ganciclovir; ES, episomal plasmid; I, possible integration signal; E, endogenous gene locus. (B) Quantitative Southern blot to analyse copy number of 5′ homology region. *dhps,* dihydropteroate synthase. P1A, P1B refer to signals resulting from hybridisation of probe 1; D, *dhps.* The ratio (P1A+P1B)/D for all clones was normalised against the ratio for wild-type 3D7 ( = 1).

In this parasite population, the plasmid is maintained largely episomally. To select for *pv1*.deletion mutants, we then applied ganciclovir pressure. Addition of this drug caused the majority of the parasites to die, however after 12 days, a ganciclovir resistant parasite population was established. We analysed this parasite population by Southern blot, using a probe diagnostic for the specific integration event. A successful double crossover integration event would result in the disappearance of the endogenous 4.1 kbp fragment, and the detection of a 5.4 kbp band. As a control, we analysed in parallel the wild-type 3D7 parental strain (Fig. 3A, lane 1), and the parasite population containing the episomally maintained knockout vector (prior to ganciclovir selection, Fig. 3A, lane 2). This experiment revealed that, despite negative selection against the presence of the plasmid backbone (and thus TK expression cassette), clonal parasites still maintain the knockout plasmid as an episome (Fig. 3A, lane 3). We were not able to detect any fragment migrating at 5.4 kbp, indicating that the *pv1* locus was still intact in these parasites.

### Drug cycling followed by negative selection allows isolation of parasites containing integrated pHTKΔPV1

Several authors have reported difficulties in negative selection using the pHTK/ganciclovir strategy to select for double crossover integration events [13], [14]. Apparently, a low copy number of episomally replicating plasmids does not always allow TK expression to a high enough level to cause parasite death following ganciclovir treatment. To negate this problem, a drug cycling strategy followed by ganciclovir selection has been suggested [14]. This appears to increase the copy number of the TK expression cassette, possibly by formation of multi-plasmid concatamers. We therefore drug cycled 3D7^TKEPI^ parasites for either 2 or 3 cycles on/off WR99210. Following drug cycling, we then added ganciclovir to select for double-crossover integration events. We monitored possible integration events using the diagnostic Southern blot detailed in the previous section. Encouragingly, parasite populations from the second and third drug cycle lost the episomal plasmid bands following ganciclovir treatment (Fig. 3A). Additionally, a fragment indicative of the correct integration event could also be detected (Fig. 3A).

### Clonal parasites show mixed genotypes and contain duplicate copies of the 5′ homology region

We cloned the parasite population resulting from 3 cycles of WR99210 selection followed by ganciclovir pressure, and analysed three independent clones, referred to as C2, C3 and E10. Diagnostic Southern blot revealed that all three clones, although apparently retaining the 4.1 kbp endogenous band, also showed a second fragment, migrating at between 5 and 6 kbp (Fig. 3A). Interestingly, whilst clones C3 and E10 showed a band at close to 6 kbp, clone C2 has obtained a fragment running at just over 5 kbp, close to the calculated size for an integration event. Nevertheless, retention of the endogenous 4.1 kbp fragment suggested that clones C2, C3 and E10 did not in fact represent a purely clonal population. For this reason, we re-cloned these parasites by limiting dilution and carried out an identical analysis to that described above. Despite this, analysis of these doubly cloned parasites still showed the presence of the same 2 specific bands when examined using Southern blot (data not shown). Such phenomena have previously been reported, and interpreted as evidence of gene duplication [13]. We corroborated this hypothesis by carrying out a quantitative Southern blot using the single copy *dhps* gene as a control. We measured signal intensity derived from Probe1 and dhps, and set the the ratio derived from wild type 3D7 to 1 as a normalisation step. In all three clones studied, the normalised ratio (total signal Probe 1)/(total signal Probe *dhps*) deviated considerably from 1, ranging from 1.8 (clone C2) to 2.5 (clone E10, Fig. 3B). Thus, clones C2, C3 and E10 contain a duplication event of at least the 5′ homology region contained on the pHTKΔPV1 plasmid.

### Despite ganciclovir resistance, clones still contain the *tk* gene. pHTKΔPV1 appears to have integrated by single crossover recombination

**Figure 4:**
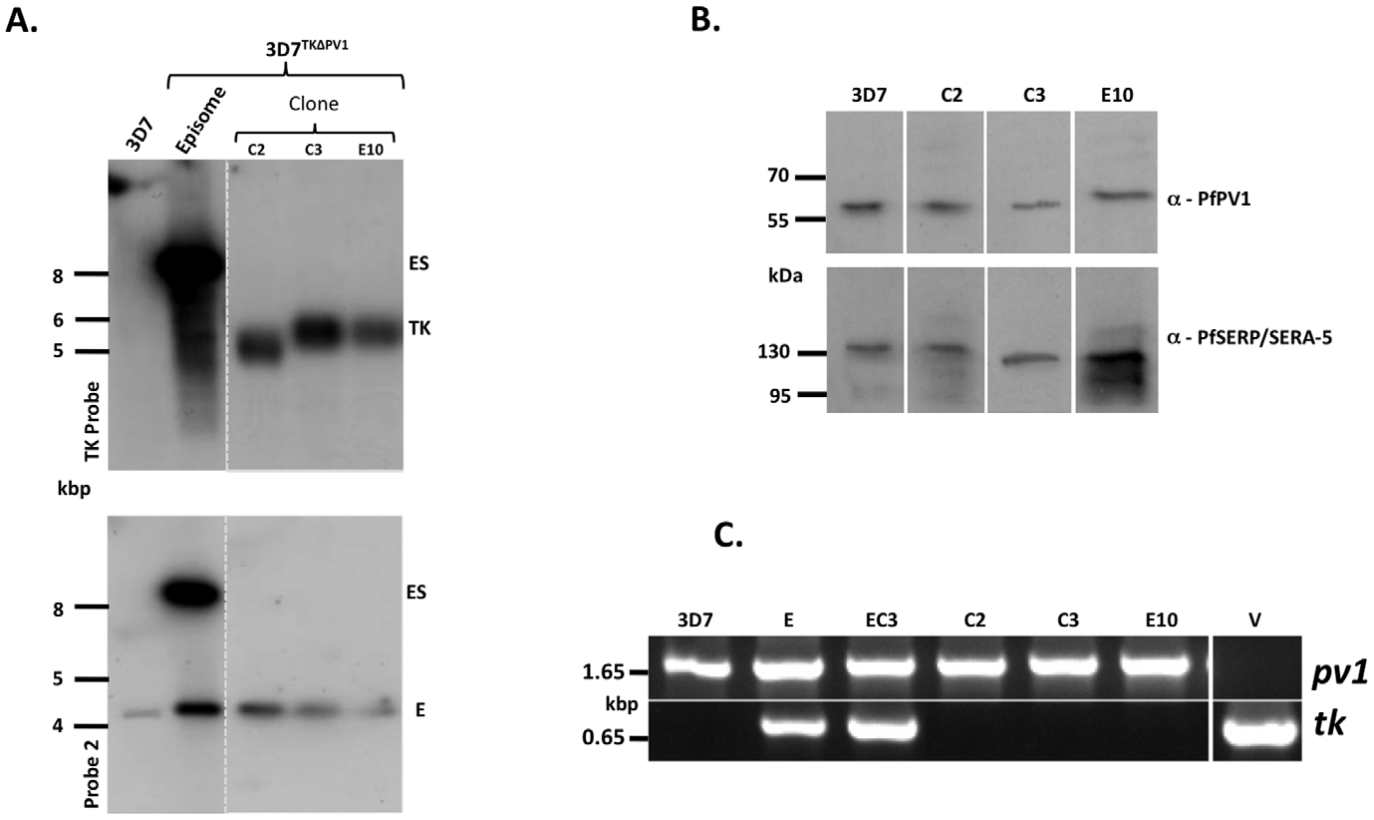
Southern blot analysis II. (A) Southern analysis on XbaI/EcoRI digested gDNA derived from the parasite lines indicated. ES, episomal plasmid; E, endogenous gene locus; TK, thymidine kinase. (B) Western blot on protein extracts derived from the clones indicated. As a loading control, we also analysed the PV resident protein PfSERA-5 (lower panel). (C) PCR analysis on gDNA extracted from the parasite clones indicated. E, parasites carrying the pHTKΔPV1 plasmid; EC3, parasites carrying the pHTKΔPV1 plasmid following 3 cycles of WR99210 selection; V, pHTKΔPV1 vector alone.

**Figure 5:**
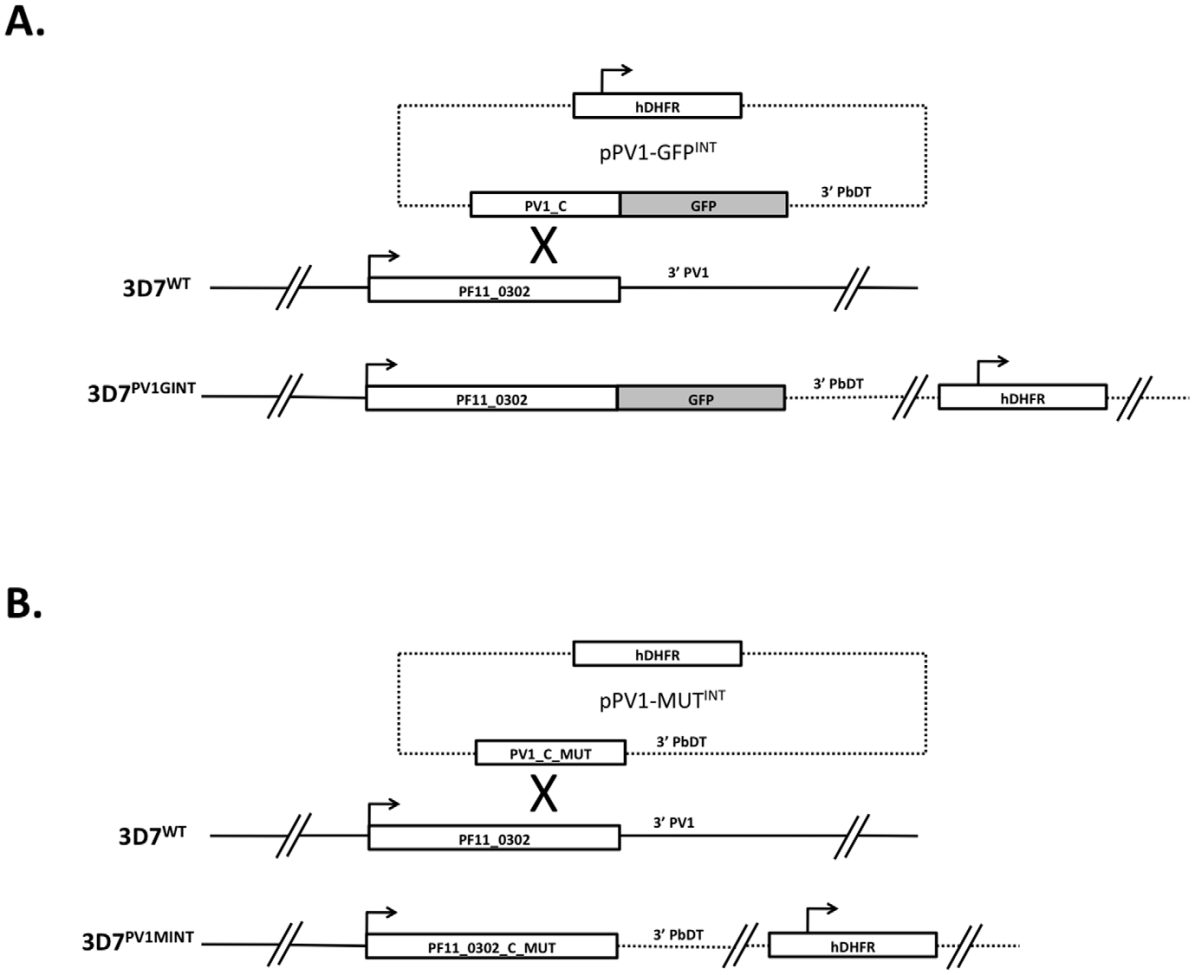
Schematic of single crossover integration strategy. (A) 3′ tagging of the endogenous *pv1* locus to generate a PfPV1-GFP chimera. (B) 3′ tagging of the endogenous *pv1* locus to regenerate a full-length *pv1* gene, but with recoding of the terminal 2 amino acids.

To understand what genetic recombination event had occurred in clones C2, C3 and E10, we carried out Southern blot analysis using two further probes. Initially, we wished to verify loss of the *tk* gene from the clonal parasites, as this would be characteristic of a double crossover integration event. Surprisingly, upon using a probe derived from the *tk* gene, we were still able to detect the tk sequence in all clones investigated, despite apparent loss of all episomally maintained plasmids (Fig. 4A). This result suggested that, despite ganciclovir pressure, the pHTKΔPV1 plasmid had integrated into the genome by single crossover, maintaining the presence of the *tk* gene. To investigate this possibility, we carried out further Southern analysis using a probe designed to be specific for the 3′ region of the integration event. In all three clones studied, this analysis revealed a banding pattern inconsistent with either a 5′ or 3′ single crossover event into the expected gene locus (Fig. 5A, S2). It is likely that the pHTKΔPV1 vector has thus integrated into a second, currently uncharacterised site within the genome. As clone C2 shows a different band pattern compared to C3 and E10 when probed with Probe 1 and the TK probe, we believe that, in this parasite line, the second site integration has occurred at a different site to that in clones C3 and E10 (Fig. 4A).

### All three clones studied contain an intact *pv1* locus, and express PfPV1, however *tk* appears recombined

All experimental evidence so far obtained strongly suggested that inactivation of the *pv1* locus had proved unsuccessful. To confirm this, we carried out both PCR and Western blot based analyses on the clonal parasite populations. Indeed, this analysis revealed that all three clones still contain a *pv1* locus of the expected size, and express PfPV1 protein to similar levels as the wild-type 3D7 parental line (Fig. 4A, upper panel, 4B). Additionally, we wished to validate the presence of a full-length copy of the *tk* gene. Although we were able to amplify the tk gene from gDNA derived from 3D7^TKEPI^ episome containing line, and the drug cycled mixed parasite population prior to ganciclovir selection, we were unable to obtain any signal for a full-length (and thus functional) *tk* gene from the clones (Fig. 4C, lower panel).

### Gene-tagging verifies the availability of the *pv1* locus for genetic manipulation

**Figure 6:**
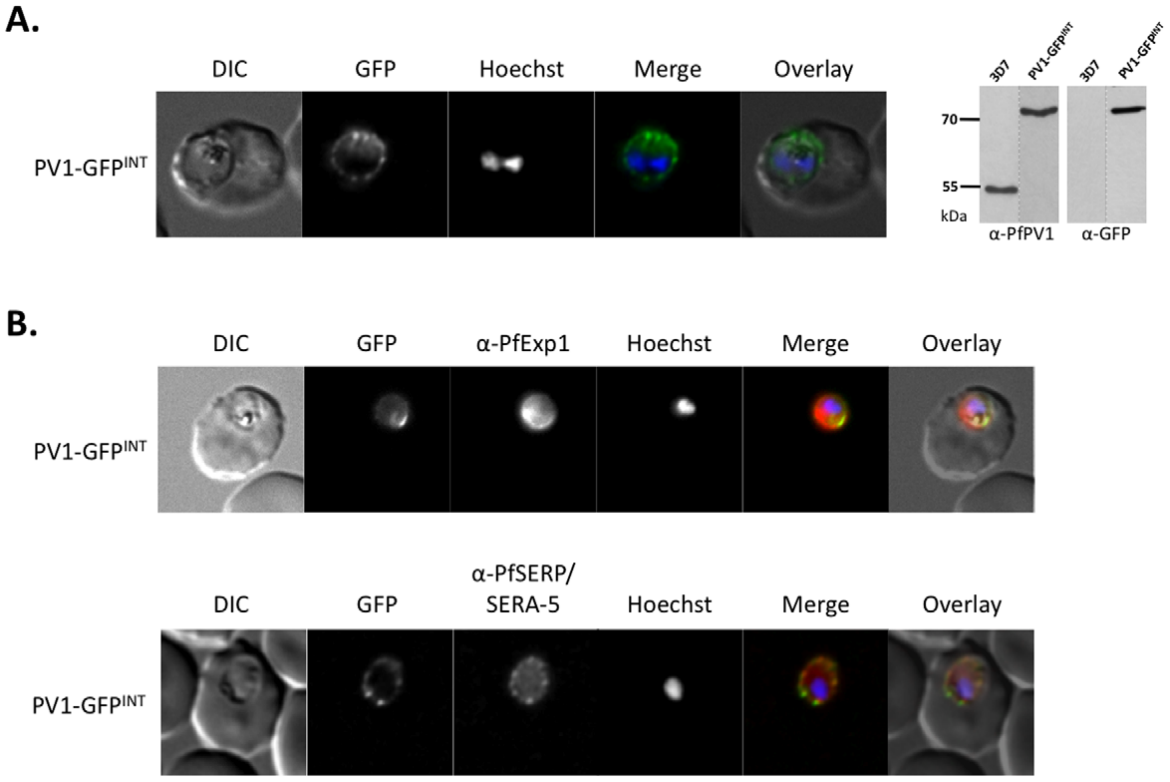
Live cell imaging. (A) Left: live cell imaging of the PV1-GFP^INT^ transgenic line; right: Western blot analysis using anti-PfPV1 anti-GFP antibodies. (B) Upper panel, colocalisation of PV1-GFP chimera with PfExp1. Lower panel, colocalisation of PV1-GFP chimera with PfSERA-5. In merge and overlay: GFP, green; specific antibody, red; Hoechst, blue.

One possible explanation for our failure to obtain *pv1* deletion mutants was that the genomic locus was not available for recombination events, thus forcing the pHTKΔPV1 plasmid to integrate into a second locus. To investigate this possibility, we designed transfection vectors for a 3′ replacement strategy, targeting the *pv1* locus. In vector pPV1-GFP^INT^, a single crossover integration event into the 3′ end of the *pv1* gene would result in a reconstituted gene locus containing the entire *pv1* gene followed by the GFP coding sequence (Fig. 5A). As the addition of GFP to the C-terminus of PV1 may have a negative influence on protein function, we also constructed pPV1-MUT, which is designed to result in a recoding of the last 2 C-terminal amino acids of PfPV1 (Fig. 5B). Both constructs were transfected into 3D7 wild-type parasites as in previous sections. 18 days after transfection, drug resistant parasite populations could be observed. These populations were then subjected to drug cycling protocols to enrich for integrants. We monitored integration of the pPV1-GFP^INT^ vector by fluorescence microscopy. After only 1 round of drug cycling, parasites could be detected that showed the expected (PV) fluorescence (Fig. 6A, left). Western blot analysis using anti-PfPV1 antisera verified loss of the circa 50kDa endogenous PfPV1 band, but the appearance of an approximately 77 kDa band, corresponding to the predicted molecular weight of the PV1-GFP chimera (Fig. 6A, right). Immunofluorescence co-localisation using antibodies against the PV and PVM markers PfSERA-5 (also referred to as SERP) and PfExp1 respectively verified that this fluorescent signal represented the PV (Fig. 6B). We isolated clones from this parasite population, and then checked the integration event by PCR on gDNA derived from individual clones. In parallel, we followed integration of the pPV1-MUT plasmid by similar means. In both cases, diagnostic PCR followed by sequencing show that pPV1-MUT, and pPV1-GFP^INT^ rapidly integrated into the *pv1* locus, in a manner consistent with a 3′ single crossover (data not shown).

## Discussion

The parasitophorous vacuole of *Plasmodium falciparum* is enriched in proteins involved in processes such as nutrient acquisition, protein trafficking and parasite egress. Although some of the PV resident proteins have homologues outside of Plasmodium spp. others, such as the PfPV1 protein herein studied, appear to be unique to the genus. This suggests that these proteins may be involved in processes particular to the intra-erythrocytic lifecycle of the parasite, and thus may be potential targets for therapeutic intervention. For this reason, we were interested in dissecting the role of PfPV1 in parasite survival. Although control experiments verified accessibility of the gene locus to genetic manipulation, multiple attempts to inactivate the *pv1* gene were unsuccessful. The lack of a dependable inducible knockout system in the *P. falciparum* system, combined with the haploid genome of asexual parasite stages makes it currently impossible to study the effect of deletion of essential genes, or those conferring a massive growth disadvantage. Thus, whilst we are unable to categorically class *pv1* as an essential gene, several lines of evidence strongly suggest this to be the case. Further analyses will be required to define the point at which these proteins are required for proper functioning of the PV.

In this current study, reverse genetics was used to investigate the importantce of PfPV1 in intra-erythrocytic survival of *P. falciparum*. Despite numerous attempts to generate *Δpv1* mutants, we were unable to isolate such parasites, although control experiments were successful. Taken together, our data strengthen the view that many proteins found in the PV perform an essential function, and highlight the importance of the PV itself for parasite survival.

## Methods

### Plasmid constructs

pPV1-GFP: The full-length PfPV1 coding sequence was amplified by RT-PCR, using the primer pair GG*CTCGAG*ATGATTAAAATAATATTAGCTAGC/GG*CCTAGG*GCTCGATATTGGTGTGTTTTGATC *(restriction sites in italics). The product was restricted with XhoI/AvrII, and inserted into similarily digested pARL2-GFP [15]. pHTKΔPV1: Fragments for gene targeting were amplified from 3D7 gDNA and introduced into the pHTK vector [12] flanking the hDHFR selection cassette. 3′ flank: GACGAATTCCGATCTCTGGAATCGGTAATGTTG/GCGCCATGGGTTTATGTAAATATATACATATAG, 5′* flank: CGG*ACTAGT*GTGATTAAGAAAAAGAATTAAAAT/CGT*AGATCT* CTATTAGTTTTGATTCTTATTATTGG (restriction sites used for cloning in italic, introduced stop codons in 5′ flank underlined). pPV1-GFP^INT^: pPV1GFP was digested with *Not*I/*Nhe*I to remove the *crt* promoter and the first 19 nucleotides of the *pv1* gene. Sticky ends filled in with Klenow (NEB) and the plasmid recircularised by T4 DNA ligase (Invitrogen). pPV1MUT^INT^: a truncated PfPV1 encoding fragment was amplified using the primer pair AT*GCGGCCGC*ACAACCAGTAACGGATTTACATG/AT*CTCGAG*TTAACTCGATATTGGTGTGTTCTGGTC (restriction sites in italics). The product was digested with *Not*I/*Xho*I, and ligated into similarly restricted pARL2 [15]. All plasmid constructs were verified by restriction digest and automatic sequencing.

### Parasite culture and transfection

The *Plasmodium falciparum* 3D7 line was cultured in human 0^+^ erythrocytes according to standard protocols, except cultures were incubated in gassed flasks [16], [17]. Transfection was carried out by electroporation of infected human 0^+^ erythrocytes as previous described [15]. GFP-transfectants were selected with 5 nM WR99210 (kindly supplied by D. Jacobus) for human DHFR- based vectors [18]. Integrant parasites were selected by repeated drug cycling (3 weeks on, 3 weeks off), and integration checked via PCR. Positive parasite populations were then cloned by limiting dilution. Integration was confirmed in each clone by integration-specific PCR, followed by sequencing to determine the exact integration site. For negative selection of pHTKΔPV1-transfected parasites, ganciclovir was added to 20 µM [12].

### Southern blotting analysis

Generally, 5 µg of genomic DNA were digested with 10 units of the appropriate restriction enzymes (NEB), separated on 0.8% agarose gels and blotted onto Hybond-N+ membrane (GE Healthcare). Membranes were probed with [α-^32^P]-dATP labelled gene specific probes following the manufacturer's protocol (HexaLabel Plus kit, Fermentas). *pv1* gene specific probes were generated using the primer pairs used to generate the pHTKΔPV1 5′ and 3′ flanks. The TK probe was amplified from the pHTK vector using the primer pair GGCCCGAAACAGGGTAAATAACG/CTTCCGAGACAATCGCGAACATC.

### Integration PCR

Single crossover integration into the 3′ end of the *pv1* gene was verified by PCR using primers ATGTGGTGGCCCCTAAGAGTG/CATTTTTACAGTTAT AAATACAATCAATTGG, followed by cloning of the PCR product and automatic sequencing (Seqlab, Göttingen).

### Western blotting

An equivalent amount of 1×10^7^ parasites were analysed by SDS-PAGE and immunoblot analysis, using mouse anti-GFP (1∶1000, Roche), and rabbit anti-PV1 (1∶500) followed by horseradish peroxidase-conjugated (HRP) anti-mouse or anti-rabbit antibody, respectively (DAKO, Santa Cruz, 1∶2000).

### IFA

IFA assays were carried out following fixation using 4% Paraformaldehyde/0.00075% Glutaraldehyde as previously described [11] except fixation was carried out at 37°C for 30 minutes, and quenching was performed with 125 mM Glycine/PBS. Alternatively, thin blood smears were allowed to dry and subsequently fixed in 100% acetone (−20°C, 10 mins). Primary antibodies used: Rabbit anti-PfPV1 (1∶500, [5]), rabbit anti-PfExp1 (1∶1000, [19]), rabbit anti-PfSERA-5 (1/1000, [20]), anti-rabbit-Cy3 (both 1/2000, DAKO) were all diluted in 3% BSA/PBS. Hoechst 33258 (Molecular probes) was used in a concentration of 50 ng/ml for fixed parasites or 10 µg/ml for live parasites. All images were acquired at either 37°C (live cells) or room temperature (fixed cells) on a Zeiss Cell Observer using appropriate filter sets. Individual images were imported into Image J64 (version 1.43b, available at http://rsb.info.nih.gov/ij), converted to 8-bit grayscale, subjected to background subtraction, and overlaid. To create figures, TIF files were imported into Powerpoint (Microsoft), assembled and slides exported as TIFs. No gamma adjustments were applied to any images, and all data is presented in accordance with the recommendations of Rossner and Yamada [21].

## Supporting Information

Figure S1 **Clustal alignment of PV1 homologues.** Predicted signal sequence is shown in grey box, PfPV1 repeat region in pink box. Only homologues from *P. knowlesi, P. falciparum* and *P. vivax* were used for this alignment, as other homologues are incomplete (see Table 1).
(TIF)

Figure S2 **Schematic of possible single crossover integration events.** 5′ CO, integration of pHTKΔPV1 via only the 5′ homology region; 3′ CO, integration of pHTKΔPV1 via only the 3′ homology region; Xb, XbaI; E, EcoRI.
(TIF)
