# Supplementary figures and images for: Genetic Evidence Strongly Support an Essential Role for PfPV1 in Intra-Erythrocytic Growth of P. falciparum
Source: PLoS One. 2011 Mar 31;6(3):e18396. doi: 10.1371/journal.pone.0018396 (PMC3069093; doi:10.1371/journal.pone.0018396)

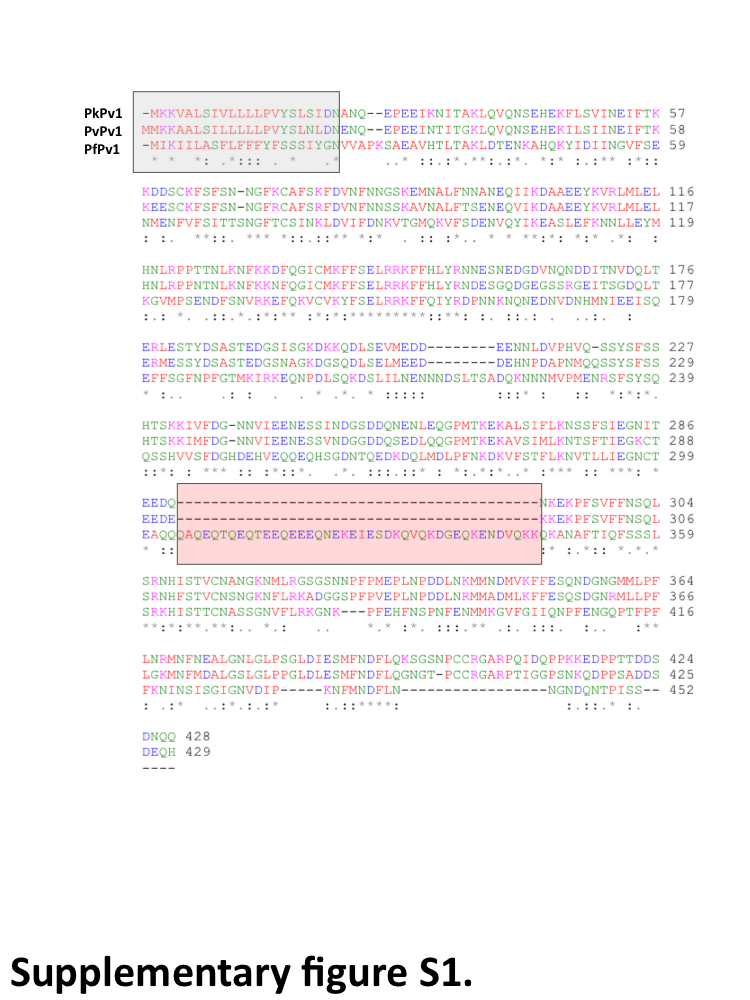

Supplement: Figure S1 — Clustal alignment of PV1 homologues. Predicted signal sequence is shown in grey box, PfPV1 repeat region in pink box. Only homologues from P. knowlesi, P. falciparum and P. vivax were used for this alignment, as other homologues are incomplete (see Table 1). (TIF) [file pone.0018396.s001.tif]

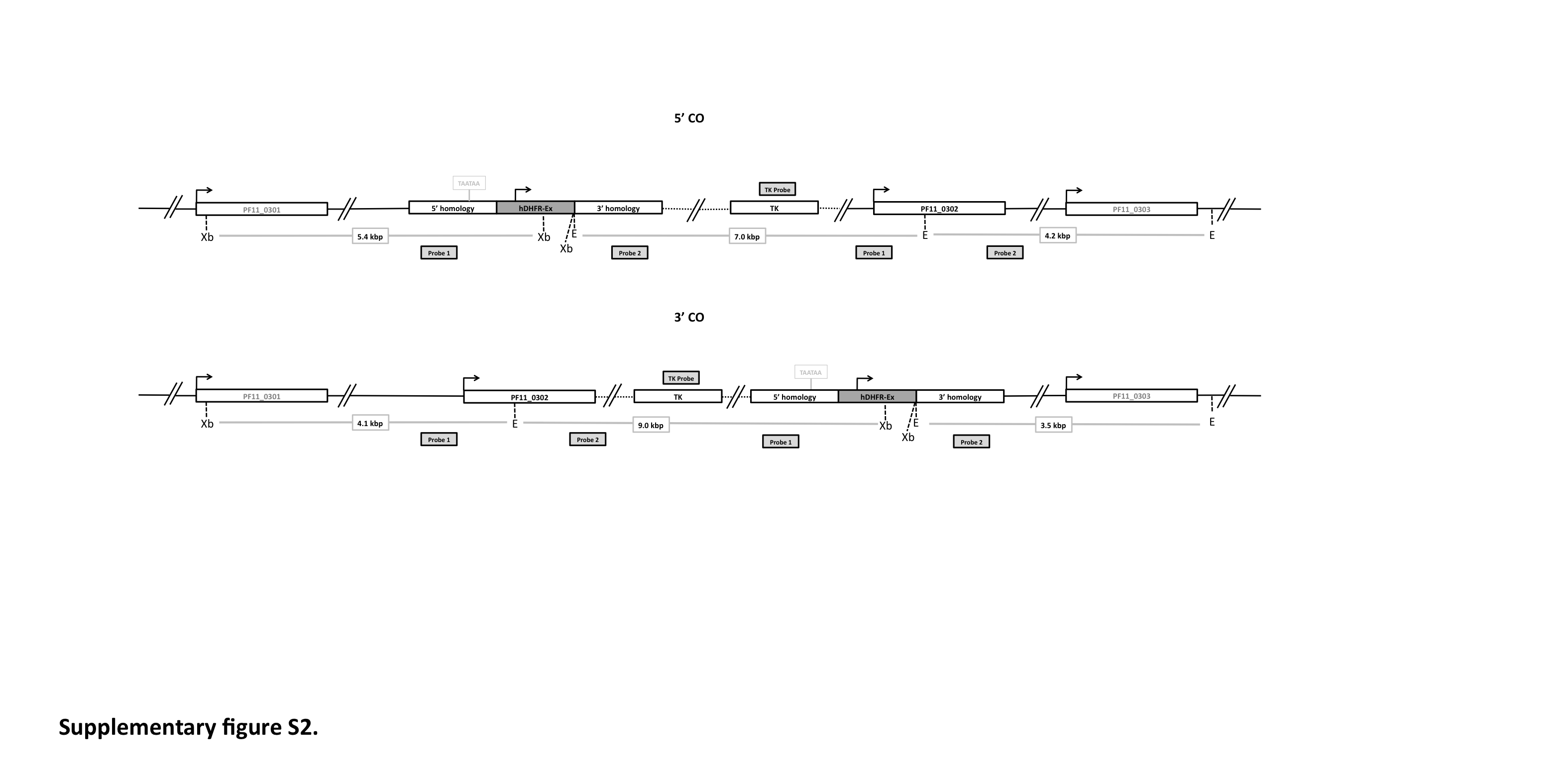

Supplement: Figure S2 — Schematic of possible single crossover integration events. 5′ CO, integration of pHTKΔPV1 via only the 5′ homology region; 3′ CO, integration of pHTKΔPV1 via only the 3′ homology region; Xb, XbaI; E, EcoRI. (TIF) [file pone.0018396.s002.tif]
